# Epidemiology and clinical characteristics of pediatric sepsis in PICUs of China: A national cross‐sectional study

**DOI:** 10.1002/mco2.211

**Published:** 2023-02-11

**Authors:** Shuang Wang, Fan Yin, Yunyu Zhang, Kang An, Yuelin Xi, Xiulan Lu, Yimin Zhu, Wugui Mo, Youpeng Jin, Dan Wei, Yumei Li, Yiyu Yang, Ying Han, Tonglin Liu, Guoping Lu, Feng Xu, Suyun Qian, Chunfeng Liu, Ying Wang, Botao Ning

**Affiliations:** ^1^ Department of Pediatric Intensive Care Unit, Shanghai Children's Medical Center Shanghai Jiaotong University School of Medicine Shanghai China; ^2^ Department of Pediatric Intensive Care Unit Hunan Children's Hospital Changsha China; ^3^ Pediatric Intensive Care Unit Hunan Provincial People′s Hospital (The First Affiliated Hospital of Hunan Normal University) Changsha China; ^4^ Department of Pediatric Intensive Care Unit Maternal and Child Health Hospital of Guangxi Zhuang Autonomous Region Nanning China; ^5^ Department of ediatric Intensive Care Unit Shandong Provincial Hospital Affiliated to Shandong First Medical University Jinan China; ^6^ Department of Pediatrics The First Affiliated Hospital of GuangXi Medical University Nanning China; ^7^ Pediatric Intensive Care Unit First Hospital of Jilin University Changchun China; ^8^ Department of Pediatric Intensive Care Unit Guangzhou Women and Children's Medical Center Guangzhou China; ^9^ Department of Pediatric Intensive Care Unit, Tongji Hospital Affiliated to Tongji Medical college Huazhong University of Science and Technology Wuhan China; ^10^ Department of Pediatric Intensive Care Unit Children's Hospital of Fudan University Shanghai China; ^11^ Department of Pediatric Intensive Care Unit Children's Hospital of Chongqing Medical University Chongqing China; ^12^ Department of Pediatric Intensive Care Unit, Beijing Children's Hospital Capital Medical University, National Center for Children's Health Beijing China; ^13^ Department of Pediatrics Shengjing Hospital of China Medical University Shenyang China

Dear Editor,

Sepsis, defined as life‐threatening organ failure induced by a dysregulated host response to infection, is a serious public health problem globally.^1^ Sepsis‐related deaths represented 19.7% of deaths globally in 2017, accounting for nearly 11 million.^2^ Approximately 1.2 million children are diagnosed with sepsis each year globally,^3^ and it is estimated that 0.521 million neonates die of sepsis yearly.^4^ Early detection of sepsis is critical for timely care and improving clinical outcomes and prognoses. As one of the most densely populated countries globally, China has the highest estimated sepsis prevalence and mortality.^5^ Furthermore, research on the prevalence, management, and clinical consequences of pediatric sepsis in China is scarce. To reveal the latest actual status of pediatric sepsis in China, we conducted the first prospective nationwide survey of pediatric sepsis.

In this study, the patients from 53 hospitals in six regions of China, who developed sepsis or newly admitted patients that met the diagnostic criteria of sepsis^6^ within the observed 24 h (From 9 AM on the third Wednesday of each month to 9 AM on the next day between December 2018 and November 2019), were enrolled in this study (Figure 1A). Patients who did not meet the selection criteria, died within 1 h of admission to the PICU, or who received extracorporeal membrane oxygenation (ECMO) or blood purification 5 days before the enrollment time were all excluded. The observation period ended if the child died, was discharged, or transferred to the general ward. The study was registered in the Chinese Clinical Trial Registry (registration number: ChiCTR1800018816).

**FIGURE 1:**
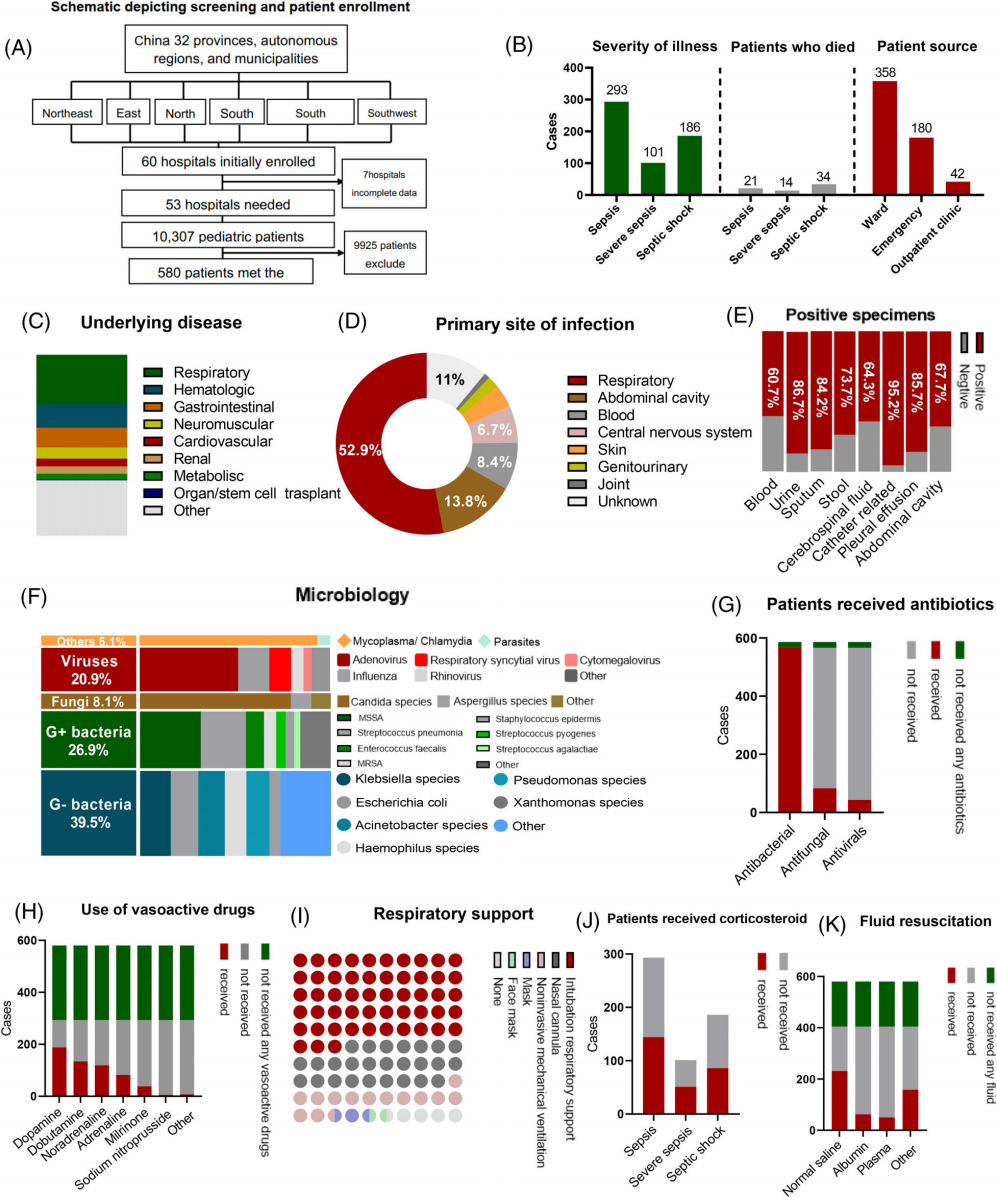
The summary of key data in this study. (A) Schematic depicting and patient enrollment. (B) Underlying disease of enrolled patients. (C) Prevalence and mortality of sepsis and patients’ source of enrolled patients. (D) The primary site of infection of sepsis patients. (E and F) Infection‐ and pathogen‐related information of enrolled patients: blood includes normal blood cultures and next‐generation sequencing (NGS), catheter related includes other invasive tubes such as intravenous catheters, indwelling urinary catheters, and tracheal intubation. Total categories do not add up to 100%, as some information from case report forms was not accurate. Cultures were derived from blood, urine, feces, abdominal effusion, pleural effusion, cerebrospinal fluid, respiratory system (nasopharynx, sputum, bronchoalveolar lavage fluid, bronchial microscope end), and skin wound. (G–K) Treatment and therapy during the observation period. (H) Included any dose of dopamine, dobutamine, noradrenaline, milrinone, sodium nitroprusside, nitroglycerin, and adrenaline. More detailed numeric data were shown in the related tables in the supplementary materials.

A total of 10,307 children in the PICU were observed during the study period, among which 580 children (57% male) met the inclusion criteria (Table S1). The overall incidence of sepsis was 5.63% (95% CI 5.2–6.1%). The distributions of patients and participating hospitals were listed in Table S2.

More than one‐third (37.8%) of these individuals had at least one underlying comorbidity, with the respiratory (24.5%) and hematological (11.6%) systems being the most often afflicted (Figure 1B). In terms of admission source, 61.5% of patients were transferred from the general ward to the PICU for advanced life support, with the remaining 31% being transported directly from the emergency department to the PICU ward. Only a tiny proportion of patients (7.2%) were seen in the outpatient department (Figure 1C). Patients with sepsis, severe sepsis, and septic shock accounted for 50.5% (293 out of 580), 17.4% (101 out of 581), and 32.0% (186 out of 580), respectively, of the total (Figure 1C). A total of 69 children died during the observation period, with total mortality of 11.9% (69 out of 580); among these, children with septic shock had the highest mortality of 18.3% (34 out of 186; 95% CI 9.4–14.8%) (Figure 1C). School‐aged children had the highest mortality (14.6%). The mortality in different age groups was shown in Table S3. According to chest X‐ray findings, only 10.3% of patients had a normal chest X‐ray. Most patients showed patchy shadows (46.9%) and lung consolidation (14.4%) (Table S1). The peripheral venous infusion was the primary drug infusion route in nearly all patients following PICU admission, with central venous catheter placement as the secondary infusion route in more than half of patients (52.1%) (Table S1).

The most common site of infection was the respiratory tract (52.9%), followed by the abdominal cavity (13.8%) (Figure 1D). Total of 760 specimens were collected from all enrolled patients, with 76.7% (583 out of 760) testing positive for an isolated infectious pathogen. The positive detection rate for blood samples was 63% (162 out of 257). Catheter specimen culture had the highest positive detection rate (100%, 65 out of 65). In all positive specimen cultures, Gram‐negative and Gram‐positive bacteria accounted for 39.5% (230 out of 583) and 26.9% (157 out of 583), respectively. Among them, *Staphylococcus aureus* (8.6%) was the most isolated bacteria, followed by *Klebsiella* species (6.3%), *Streptococcus pneumonia* (6.3%), and *Escherichia coli* (5.7%). The rate of fungi, viruses, and parasites isolated was of 8.1, 20.9, and 0.3%, respectively (Figures 1E and F). More detailed numeric data about infection and pathogen were shown in Table S4.

Regarding treatment, 100% of patients were treated with antibacterial, 7.6% with antivirals, and 14.7% with antifungal agents (Figure 1G). Noninvasive mechanical ventilation was used in 13.3% of patients and mechanical ventilation respiratory support was applied in 52.8% patients (Figure 1I). Corticosteroid infusion was also commonly used in severe sepsis (51 out of 101, 50.5%), followed by sepsis (144 out of 293, 49.1%) and septic shock (86 out of 186, 46.2%). Totally 69.7% of patients received fluid resuscitation, with normal saline (57.2%) being the most common choice, followed by albumin (15.3%) (Figure 1J). Dopamine was the most used (64.2%) in children who received vasoactive drug therapy. This was followed by dobutamine of 46% and norepinephrine of 41% (Figure 1H). Detailed numeric data about treatment and therapy were shown in Table S5. The most common complication was multiple organ failure (*n* = 197, 34.9%), followed by acute gastrointestinal injury (*n* = 176, 30.3%) and sepsis‐associated encephalopathy (*n* = 147, 25.3%) (Table S6).

This extensive, nationwide, multicenter cross‐sectional study of 10,307 children in 53 hospitals reveals that pediatric sepsis is still quite common in China, with a PICU sepsis prevalence of 5.6% and high septic shock mortality (18.3%). Although our reported prevalence was lower than the estimated incidence of childhood sepsis in Asia (15.3%),^7^ this incidence was adequate to demonstrate that sepsis was still a severe health danger to Chinese children currently. Our results were consistent with most studies' observations, with respiratory and hematologic/immune disorders being the most prevalent main conditions.^4^ In our study, Gram‐negative bacteria were most frequently isolated pathogens (39.5%), which was consistent with global ICU infection prevalence data.^8^ Jabornisky et al.^9^ reported that Gram‐negative bacteria were the most common pathogens, accounting for 36.6% of children with sepsis in Argentina. Contrary to our findings, several observational studies from the United States suggest that Gram‐positive bacteria predominate in the pathogenesis of sepsis.^8^ Gram‐negative bacteria are closely associated with healthcare‐associated infection (catheter‐ or ventilator‐associated infection, opportunistic infection), highlighting the need to reinforce infection control and routine monitoring of in‐hospital epidemiology. The SPROUT^7^ had concluded that sepsis patients transferred to the PICU for continued treatment from an inpatient unit were in worse underlying conditions and had greater probability of comorbidities, so this category required more care than sepsis patients transferred from emergency or outpatient setting. Additional research is required to determine whether there are statistical differences in the types of severity, pathogens, and prognosis among patients from different sources. Nearly half of the patients in this study were treated with glucocorticoids, especially in sepsis group. However, the effectiveness of glucocorticoids for the treatment of sepsis is still unclear till now. Meanwhile, the usage of glucocorticoids was considered to be an independent risk factor for death in some reports.^10^ Although we could not rule out the potential whether the use of glucocorticosteroids increased or decreased mortality, our findings indicated that there was a considerable readiness to use glucocorticosteroids clinically in China at present. Therefore, even as enthusiasm for glucocorticoids in sepsis wanes, there remains a need for adequate clinical trials to determine the efficacy of glucocorticoids and optimize the timing of its therapy for pediatric sepsis in the future study.

To sum up, till now, deficiencies remain in the diagnosis and treatment of sepsis in China. Sepsis remains high prevalence and mortality. Evidence using epidemiological data will improve the understanding and management of sepsis and the application of relevant septic guidelines in the future, which are essential for improving the prognosis of pediatric sepsis.

## AUTHOR CONTRIBUTIONS

B. T. N., Y. W., C. F. L., and S. Y. Q. designed the study. All the participant hospitals contributed to the collection of data. S. W., F. Y., and Y. Y. Z. analyzed the data. S. W., Y. Y. Z., F. Y., and B. T. N. wrote the manuscript. B. T. N., S. W., F. Y., Y. Y. Z., K. A., and Y. W. discussed and interpreted the results. Y.L.X., X. L. L., Y. M. Z., W. G. M., Y. P. J., D. W., Y. M. L., Y. Y. Y., Y. H., T. L. L., G. P. L., F. X., S. Y. Q., C. F. L., and Y. W. were the representatives of the top 13 hospitals in terms of number of the collected cases. All the name of participant hospitals were listed in Supplementary Materials. All authors contributed to the article and approved the submitted version.

## CONFLICT OF INTEREST

The authors declare no conflicts of interest to report.

## FUNDING STATEMENT

This work was supported by the National Key Research and Development Program of China (2021YFC2701800, 2021YFC2701805), the Shanghai Municipal Commission of Health and Family Planning (2016ZB0104), the Shanghai Natural Science Foundation of China (19ZR1432900) and the Shanghai Translational Medicine Collaborative Innovation Center (TM202012).

## ETHICS STATEMENT

This study was approved by the ethical committee of Shanghai Children's Medical Center (SCMCIRB‐K2018030).

## Supporting information

Supporting Information

## Data Availability

The datasets generated during and/or analyzed during the current study are available from the corresponding author on reasonable request.
