# Supplementary material for: Epidemiology and clinical characteristics of pediatric sepsis in PICUs of China: A national cross‐sectional study
Source: MedComm (2020). 2023 Feb 11;4(1):e211. doi: 10.1002/mco2.211 (PMC9921814; doi:10.1002/mco2.211)
Supplement: Supplementary file 1 — Supporting Information [file MCO2-4-e211-s001.pdf]

Supplementary Materials

Epidemiology and clinical characteristics of pediatric sepsis in PICUs of China: a national cross-sectional study

Shuang Wang[1#], Fan Yin[1#], Yunyu Zhang[#1], Kang An[1], Yuelin Xi[1], Xiulan Lu[2], Yimin Zhu[3], Wugui Mo[4], Youpeng Jin[5], Dan Wei[6], Yumei Li[7], Yiyu Yang[8], Ying Han[8], Tonglin Liu[9], Guoping Lu[10], Feng Xu[11], Suyun Qian*[12], Chunfeng Liu*[13], Ying Wang*[1], Botao Ning*[1]

[1]Department of Pediatric Intensive Care Unit, Shanghai Children's Medical Center, Shanghai Jiaotong University School of Medicine, Shanghai, China

[2]Department of Pediatric Intensive Care Unit, Hunan Children's Hospital, Changsha, China

[3]Department of Pediatric Intensive Care Unit, Hunan Provincial People's Hospital (The First Affiliated Hospital of Hunan Normal University), Changsha, China

[4]Department of Pediatric Intensive Care Unit, Maternal and Child Health Hospital of Guangxi Zhuang Autonomous Region, Nanning, China

[5]Department of Pediatric Intensive Care Unit, Shandong Provincial Hospital Affiliated to Shandong First Medical University, Jinan, China

[6]Department of Pediatrics, The First Affiliated Hospital of GuangXi Medical University, Nanning, China

[7]Department Pediatric Intensive Care Unit, First Hospital of Jilin University, Changchun, China

[8]Department of Pediatric Intensive Care Unit, Guangzhou Women and Children's Medical Center, Guangzhou, China

[9]Department of Pediatric Intensive Care Unit, Tongji Hospital Affiliated to Tongji Medical college, Huazhong University of Science and Technology, Wuhan, China

[10]Department of Pediatric Intensive Care Unit, Children's Hospital of Fudan University, Shanghai, China

[11]Department of Pediatric intensive Care Unit, Children's Hospital of Chongqing Medical University, Chongqing, China

[12]Department of Pediatric Intensive Care Unit, Beijing Children's Hospital, Capital Medical University, National Center for Children's Health, Beijing, China

[13]Department of Pediatrics, Shengjing Hospital of China Medical University, Shenyang, China

* Correspondence:

Botao Ning, Department of Pediatric intensive care unit, Shanghai Children's Medical Center, Shanghai Jiaotong University School of Medicine, Shanghai 200127, China.

Email: ningbotao@126.com

Yin Wang, Department of Pediatric intensive care unit, Shanghai Children's Medical Center, Shanghai Jiaotong University School of Medicine, Shanghai 200127, China.

Email: ywang_picu@shsmu.edu.cn

Chunfeng Liu, Department of pediatrics, Shengjing Hospital of China Medical University, Shenyang 110004, China.

Email: zhliu258@hotmail.com

Suyun Qian, Pediatric intensive care unit, Beijing Children's Hospital, Capital Medical University, National Center for Children's Health, Beijing 100045, China.

Email:syqian2020@163.com

[#]Shuang Wang, Fan Yin and Yunyu Zhang contributed equally to this study.

Study design and participants

This was a cross-sectional study investigating the prevalence, treatment, and outcomes for pediatric patients with sepsis admitted to the PICU. The study period included 12 days over the course of 1 year: December 19, 2018, and January 16, February 13, March 13, April 17, May 15, June 17, July 17, August 14, September 18, October 16, and November 13 in 2019. All enrolled hospitals participated voluntarily in this survey, and written informed consent was obtained from children's legal guardians. Seven hospitals were excluded due to incomplete information records. Overall, a total of 580 children from 53 hospitals in 6 regions of China were enrolled (shown in Figure S1). We collected demographic, microbiological, therapeutic and prognostic data from all included pediatric patients newly diagnosed as sepsis. All enrolled hospitals were equipped with a separate intensive care unit only for critically ill pediatric patients. Hospitals were evenly distributed in different regions across mainland China, which is sufficiently representative to reflect the current status of pediatric sepsis nationwide. This study was approved by the ethical committee of Shanghai Children's Medical Center (SCMCIRB-K2018030) and was registered in the Chinese Clinical Trial Registry (registration number: ChiCTR1800018816).

Inclusion and exclusion criteria

All children aged 28 days (corrected gestational age >41 weeks for premature infants) to 18 years, regardless of sex, who had a diagnosis of sepsis, severe sepsis, or septic shock were included. Definitions were based on the 2005 International Pediatric Sepsis Consensus Criteria[1], and listed as follows: (1) sepsis: presence of two or more signs of systemic inflammatory response syndrome (SIRS; one of which must be abnormal temperature or leukocyte count) with suspected or potential infection; (2) severe sepsis: sepsis plus acute respiratory distress syndrome or dysfunction of two or more organs (neurological, hepatic, hematological, or renal); (3) septic shock: sepsis plus cardiovascular organ dysfunction. In this study, we only included patients with sepsis who were newly diagnosed within the observed 24 hours (From 9 AM on the third Wednesday of each month to 9 AM on the next day between December 2018 and

November 2019). This includes patients in the ICU who developed sepsis or newly admitted patients that meet the diagnostic criteria of sepsis within the observed 24 hours, excluding previously diagnosed patients with sepsis. Case information was recorded only if patients met the diagnostic criteria for sepsis within this 24-hour time window. To calculate the point prevalence of sepsis, we collected the total number of patients in the PICU on the study day, even if there were no newly diagnosed sepsis cases. Patients who did not meet the selection criteria, died within 1 hour of admission to the PICU, or who received extracorporeal membrane oxygenation (ECMO) or blood purification 5 days before the enrollment time, were all excluded. The observation period ended if the child died, was discharged, or transferred to the general ward.

Data collection

With the help of data engineers, an internet (http://101.231.181.227:8088) was used to collect data voluntarily uploaded by trained clinicians from the enrolled hospitals through google browser. Standardized case report forms (CRFs) were specially designed for data collection. All CRFs were assigned a unique identifier. Once patients were diagnosed with sepsis, their relevant medical information was recorded in the CRFs. Direct supervision for completing the CRFs was provided for remote regions through telephone and email, to ensure that all CRFs were completed in a standardized manner. Once the data were uploaded, the backstage administrator could review all the original information and contact the site coordinators if there was any error in completing the CRFs.

Statistical analyses

We performed a descriptive analysis of the current epidemiological status of pediatric sepsis in mainland China. Prevalence rate was used to estimate the incidence of sepsis in PICUs nationwide. This was calculated by considering all newly diagnosed patients with sepsis (incident cases) on the study day, divided by the total number of PICU patients observed in the study period. Regional and national prevalence rates were calculated. Categorical data are expressed as cases (%). All statistical analysis was performed using IBM SPSS Statistics 25 (IBM Corp., Armonk, NY, USA). The mortality rate was calculated as the number of all deaths divided by the total patients

with sepsis and presented with the 95% confidence interval (CI).

Study investigators

Northwest

Qinghai Provincial Women and Child Health Hospital

Xianyang Children's Hospital

Xi'an Children's Hospital

Baoji Children's Hospital

Northwest Women's and Children's Hospital

The First Affiliated Hospital of Xinjiang Medical University

General Hospital of Ningxia Medical University

The First Hospital of Lanzhou University

East

Jiangxi Provincial Children's Hospital

Shanghai Children's Medical Center

Children's Hospital of Fudan University

Anhui Provincial Children's Hospital

The Children's Hospital of Zhejiang University School of Medicine

Children's Hospital of Shanghai

Shandong Provincial Hospital

Children's Hospital of Nanjing Medical University

Xinhua Hospital Affiliated to Shanghai Jiaotong University School of Medicine

Children's Hospital of Soochow University

The 2nd School of Medicine, WMUW/The 2nd Affiliated Hospital and Yuying Children's Hospital

South central

The First Affiliated Hospital, Sun Yat-sen University

Guangzhou Women and Children's Medical Center

The First Affiliated Hospital of Guangxi Medical University

Wuhan Children's Hospital, Wuhan Maternal and Child Healthcare Hospital

Henan Children's Hospital

Hunan Children's Hospital

Tongji Medical College of Hust

The First Affiliated hospital of Zhengzhou University

Hunan Provincial People's Hospital

Hainan Women and Children's Medical Center

Shenzhen Children's Hospital

Maternity and Child Health Care of Guangxi Zhuang Autonomous Region

Northeast

Shengjing Hospital of China Medical University

The First Bethune Hospital of Jilin University

Children's Hospital of Changchun

Harbin Children's Hospital

Dalian Children's Hospital

Mudanjiang Children Hospital

North

Beijing Children’s Hospital

Tianjin Children's Hospital

Baoding Children's Hospital

The First People's Hospital of Datong

Children's Hospital of Shanxi

Hebei children's hospital

Children’s Hospital Capital Institute of Pediatrics

Inner Mongolia People's Hospital

The Affiliated Hospital of inner Mongolia Medical University

Southwest

Tibet Autonomous Region People's Hospital

West China Second University Hospital, Sichuan University/West China Women's and Children's Hospital

Kunming Children's Hospital

Chengdu Women's and Children's Central Hospital

Guizhou Provincial People's Hospital

Children's Hospital of Chongqing Medical University

Maternal and Child Health Hospital of Guiyang Province

References:

1. Goldstein B, Giroir B, Randolph A, International Consensus Conference on Pediatric Sepsis. International pediatric sepsis consensus conference: definitions for sepsis and organ dysfunction in pediatrics. Pediatr Crit Care Med. 2005;6(1):2-8.

Supplementary Figure and Tables

TABLE S1 | Baseline information of enrolled patients

Characteristic	N	n%
Nationality		
Han nationality	519	89.5 (519/580)
Ethic minority	61	10.5 (61/580)
Sex		
Male	330	57.0 (330/580)
Female	250	43.0 (250/580)
Underlying diseases		
Respiratory	142	24.5 (142/580)
Hematologic/immune system	67	11.6 (67/580)
Gastrointestinal	57	9.8 (57/580)
Neuromuscular	32	5.5 (32/580)
Cardiovascular	23	3.9 (23/580)
Renal	21	3.6 (21/580)
Inborn errors of metabolism	17	2.9 (17/580)
Organ/stem cell transplant	3	0.5 (3/580)
Other	159	27.4 (159/580)
Severity of illness		
Sepsis	293	50.5 (293/580)
Severe sepsis	101	17.4 (101/580)
Septic shock	186	32.0 (186/580)
Patients who died	69	11.9 (69/580)
Sepsis	21	7.2 (21/293)
Severe sepsis	14	13.8 (14/101)
Septic shock	34	18.3 (34/186)
Patient source		
Ward	358	61.7 (358/580)
Emergency	180	31.0 (180/580)
Outpatient clinic	42	7.2 (42/580)
Chest X-ray findings		
Patchy shadow	272	46.9 (271/580)
Lung consolidation	84	14.4 (84/580)
Normal	60	10.3 (60/580)
Pleural effusion	46	7.9 (46/580)
Air bronchogram	10	1.7 (10/580)
Pneumothorax	1	0.2 (1/580)
Atelectasis	7	1.2 (7/580)
Lung abscess	8	1.4 (8/580)
Other	83	14.3 (83/580)
Unknown	9	1.7(9/580)
Infusion route[a]		

Continued table S1

First infusion route		
Bone marrow	2	0.3(2/574)
Peripheral venous	526	91.6(526/574)
Central venous	46	8.0(46/574)
Second infusion route		
Bone marrow	0	0
Peripheral venous	271	47.2(271/574)
Central venous	303	52.8(271/574)

[a] Excluded 6 items of unclear data.

TABLE S2 | Regional distribution, population, and prevalence of sepsis

Region	Included patients	Observed patients	Hospitals	Total prevalence
Northwest	46	927	8	4.96%
East	117	2211	11	5.29%
South Central	279	2852	12	9.78%
Northeast	56	1318	6	4.25%
North	44	788	9	5.58%
Southwest	38	953	7	3.99%
Total	580	10307	53	5.63%

TABLE S3 | Age groups among patients who died

Age groups (years)	Total patients	Patients who died	Mortality (%)
Infancy (0–1)	321	39	12.1
Toddler (1–3)	106	11	10.4
Preschool age (3–6)	59	6	10.2
School age (6–15)	89	13	14.6
Adolescent (15–18)	2	0	0

TABLE S4 | Infection- and pathogen-related information of enrolled patients

Category	N	(%)
Primary site of infection		
Respiratory	307	52.9 (307/580)
Abdominal cavity	80	13.8 (80/580)
Blood	49	8.4 (49/580)
Central nervous system	39	6.7 (39/580)
Skin	23	4.0 (23/580)
Genitourinary	12	2.1 (12/580)
Joint	6	1.0 (6/580)
Unknown	64	11.0 (64/580)
Positive number of different source of specimens		
Blood[a]	162	63.0 (162/257)
Sputum	242	84.6 (242/286)
Urine	26	86.7 (26/30)
Stool	14	71.4(14/21)
Cerebrospinal fluid	18	64.3 (18/28)
Pleural effusion	18	85.7 (18/21)
Abdominal cavity	21	72.4(21/29)
Catheter related[b]	65	100 (65/65)
Bone marrow	5	100(5/5)
Microbiologyc		
Total cultures with positive isolated	583[c]	76.7 (583/760)
Gram-negative bacteria	230	39.5 (221/583)
Klebsiella species	37	6.3 (37/583)
Escherichia coli	33	5.7 (33/583)
Acinetobacter species	32	5.5 (32/583)
Haemophilus species	26	4.5 (26/583)
Pseudomonas species	28	4.8 (28/583)
Xanthomonas species	13	2.2 (13/583)
Other	61	10.5 (52/583)
Gram-positive bacteria	157	26.9 (157/583)
Methicillin-sensitive Staphylococcus aureus	50	8.6 (50/583)
Streptococcus pneumonia	37	6.3 (37/583)
Enterococcus faecalis	15	2.6 (15/583)
Methicillin-resistant Staphylococcus aureus	10	1.7 (10/583)
Staphylococcus epidermis	8	1.4 (8/583)
Streptococcus pyogenes	7	1.2 (7/583)
Streptococcus agalactiae	5	0.9 (5/583)
Other	25	4.3 (25/583)
Fungi	47	8.1(47/583)
Candida species	37	6.3 (32/583)
Aspergillus species	5	0.9 (5/583)
Other	5	0.9 (9/583)

Continued table S4

Viruses	122	20.9(122/583)
Adenovirus	63	10.8 (63/583)
Influenza	20	3.4 (20/583)
Respiratory syncytial virus	14	2.4 (14/583)
Rhinovirus	8	1.4 (8/583)
Cytomegalovirus	5	0.9 (5/583)
Other	12	2.0 (12/583)
Mycoplasma/ chlamydia	28	4.8 (28/583)
Parasites	2	0.3 (2/583)

[a] Blood includes normal blood cultures and Next Generation Sequencing (NGS);

[b] Catheter related includes other invasive tubes such as intravenous catheters, indwelling urinary catheters, and tracheal intubation;

[c] Some samples with more than one pathogen detected, therefore the total number of pathogens was higher than the number of positive samples;

[d] Total categories do not add up to 100%, as some information from case report forms was not accurate;

[e] Cultures were derived from blood, urine, feces, abdominal effusion, pleural effusion, cerebrospinal fluid, respiratory system (nasopharynx, sputum, bronchoalveolar lavage fluid, bronchial microscope end), and skin wounds.

TABLE S5 | Treatment and therapy during the observation period

Category	N	(%)
Number of patients received antibiotics	566	97.6 (566/580)
Antibacterial	566	100 (566/566)
Antifungal	83	14.7 (83/566)
Antivirals	43	7.6 (43/566)
Vasoactive drugs[a]		
Patients received vasoactive drugs	293	50.5 (293/580)
Dopamine	188	64.2 (188/293)
Dobutamine	134	45.7 (134/293)
Noradrenaline	119	40.6 (119/293)
Adrenaline	82	28.0 (82/293)
Milrinone	38	13.0 (38/293)
Sodium nitroprusside	5	1.7 (5/293)
Nitroglycerin	0	0
Other	7	2.4 (7/293)
Number of vasoactive drugs used		
1	137	46.8 (137/293)
2	90	30.7 (90/293)
3	50	17.1 (50/293)
4	11	3.8 (11/293)
>4	3	1.0 (3/293)
Respiratory support		
Intubation respiratory support	306	52.8 (306/580)
Nasal cannula	153	26.4 (153/580)
Noninvasive mechanical ventilation	77	13.3 (77/580)
Mask	13	2.2 (13/580)
Face mask	5	0.9 (5/580)
None	26	4.5 (26/580)
Patients received corticosteroid	281	48.4 (281/580)
Sepsis	144	49.1 (144/293)
Severe sepsis	51	50.5 (51/101)
Septic shock	86	46.2 (86/186)
Fluid resuscitation	404	69.7 (404/580)
Normal saline	231	57.2 (231/404)
Albumin	62	15.3 (62/404)
Plasma	50	12.4 (50/404)
Other	158	39.1 (158/404)
Types of resuscitation fluid used		
1	320	79.2 (320/404)
2	70	17.3 (70/404)
3	14	3.5 (14/404)
Albumin[b]	306	52.6 (306/580)
Artificial colloid	30	5.2 (30/580)

Continued table S5

Blood transfusion	341	58.8 (341/580)
Enteral nutrition	386	66.6 (386/580)
Parenteral nutrition	228	39.3 (228/580)
Prevention of stress ulcer	233	40.2 (233/580)
Insulin	25	4.3 (25/580)
Intravenous gamma globulin	328	56.6 (328/580)
Continuous renal replacement therapy	101	17.4 (101/580)
Plasma exchange	46	7.9 (46/580)
Extracorporeal membrane oxygenation	6	1.0 (6/580)

[a] Included any dose of dopamine, dobutamine, noradrenaline, milrinone, sodium nitroprusside, nitroglycerin, and adrenaline；

[b] Includes albumin used for fluid resuscitation and for supporting therapies such as for hypoalbuminemia.

TABLE S6 | Complications in patients with sepsis.

Complications	N	n (%)
Multiple organ dysfunction syndrome	197	34.9 (197/580)
Acute gastrointestinal injury	176	30.3 (176/580)
Sepsis-associated encephalopathy	147	25.3 (147/580)
Acute respiratory distress syndrome	113	19.5 (113/580)
Acute kidney injury	103	17.8 (103/580)
Sepsis-induced myocardial dysfunction	81	14.0 (81/580)
Disseminated intravascular coagulation	72	12.4 (72/580)
Deep vein thrombosis	24	4.1 (24/580)
Abdominal compartment syndrome	18	3.1 (18/580)
None	181	31.15 (181/580)
